# Effects of Chlorogenic Acid on Cellular Senescence in an In Vitro Model of 3T3-L1 Murine Adipocytes

**DOI:** 10.3390/molecules31010167

**Published:** 2026-01-01

**Authors:** Maria Sofia Molonia, Federica Lina Salamone, Santi Trischitta, Antonella Saija, Francesco Cimino, Antonio Speciale

**Affiliations:** 1 Department of Chemical, Biological, Pharmaceutical and Environmental Sciences, University of Messina, Viale F. Stagno D’Alcontres 31, 98166 Messina, Italy; mmolonia@unime.it (M.S.M.); federica.salamone@studenti.unime.it (F.L.S.); santi.trischitta@studenti.unime.it (S.T.); asaija@unime.it (A.S.); specialea@unime.it (A.S.); 2 “Prof. Antonio Imbesi” Foundation, University of Messina, 98100 Messina, Italy

**Keywords:** cellular senescence, chlorogenic acid, adipose tissue dysfunction, 3T3-L1 cells

## Abstract

Cellular senescence is a stress-induced process that contributes to adipose tissue dysfunction by promoting inflammation, impaired adipogenesis, and insulin resistance, alterations that are closely associated with age-related cellular dysfunction and metabolic disorders. In this study, we evaluated the protective role of chlorogenic acid (CGA), a polyphenol with known antioxidant and anti-inflammatory properties, against oxidative stress-induced senescence in murine 3T3-L1 adipocytes. The results obtained showed that CGA treatment significantly alleviated the senescent phenotype by restoring Lamin B1 levels and the Bcl-2/Bax ratio. Additionally, CGA downregulated key senescence-related cell cycle progression markers, modulating p53, p21, and MAPK signaling. CGA also restored insulin signaling through the PI3K-AKT-GLUT4 axis and improved glucose uptake, while attenuating oxidative stress, inflammatory cytokine expression, and extracellular matrix remodeling factors associated with SASP. Collectively, these findings support the role of CGA as a promising senotherapeutic nutraceutical able to reduce adipocyte senescence and its metabolic consequences, offering novel insights for the development of dietary supplements targeting age-related cellular dysfunction.

## 1. Introduction

Adipose tissue plays an important role as an organ in which energy is stored in the body. For the maintenance of lipid homeostasis and energy balance, a crucial role is played by adipocytes [1]—cells that differentiate from preadipocytes during the adipogenesis process and have the ability to store triacylglycerols or release free fatty acids in response to energy requests [2]. However, adipose tissue is not only a storage organ but also constitutes the largest endocrine organ in the body and, as such, participates in maintaining its homeostasis [3]. In fact, adipocytes actively secrete various bioactive molecules, known as adipokines, which influence energy homeostasis and play important roles in both physiological and pathological processes [4].

Adipose tissue dysfunction occurs in obesity when a continuous excess of nutrients results in the impairment of the adipogenesis process [5], and it is accompanied by local and systemic chronic low-grade inflammation, linked to the development of insulin resistance, metabolic syndrome, and cardiovascular diseases [6]. A contribution to this condition of adipose tissue dysfunction appears to be given by the accumulation of senescent cells, reported in the context of aging, obesity, and diabetes [7].

Senescence is a condition that can affect cells in many different tissues of the body, whether stem and progenitor cells or terminally differentiated cells. It is induced by sustained DNA damage that arises from either replicative or oxidative stress, and it is mainly characterized by permanent cell cycle arrest and the development of a senescence-associated secretory phenotype (SASP), with the active release of an array of pro-inflammatory cytokines, chemokines, and extracellular matrix remodeling factors [8,9]. Both mature adipocytes and their precursors—preadipocytes—can undergo senescence phenomena, induced both by aging and by conditions of oxidative stress. Senescent adipocytes exhibit a SASP profile characterized by the increased release of cytokines such as IL-6, IL-8, IL-1α, IL-1β, TNF-α, and matrix metalloproteinases MMP-3 and MMP-12 [8] contributing to local tissue inflammation and macrophage infiltration. Furthermore, through the effects of mediators released by SASP, senescence not only affects the proliferation and differentiation of preadipocytes but also induces senescence in neighboring cells, causing the dysfunction of other cells present in the tissue [10], with negative impacts on adipogenesis as well as adipose tissue insulin sensitivity, which can, in the long run, promote the development of type 2 diabetes [11].

In light of this, and given that the accumulation of senescent cells has been observed in several pathologies [7], in recent years, research has focused on molecules capable of counteracting them, called senotherapeutics. These drugs can act through two mechanisms. Senolytic agents act directly on senescent cells by selectively killing them by inducing apoptosis, whereas senomorphics act by modulating SASP and thus suppressing the related inflammation without eliminating senescent cells [9,11]. In this context, numerous dietary natural compounds possess the ability to act as senotherapeutic agents. Polyphenols, in particular, are molecules with antioxidant, radical scavenger, and anti-inflammatory properties and are therefore the focus of research in this field. Studies have reported the senotherapeutic activity of several polyphenols for which senolytic (e.g., quercetin) or senomorphic (e.g., resveratrol, apigenin, kaempferol, luteolin, wogonin) activity or both (e.g., fisetin, epigallocatechin gallate, curcumin) has been demonstrated [12,13].

Our interest lies in chlorogenic acid (5-O-caffeoyl-quinic acid; CGA), a dietary polyphenol endowed with antioxidant and anti-inflammatory properties, showing antiadipogenic and antidiabetic potential [14], which has been reported to counteract oxidative stress and age-related cellular dysfunction [15]. CGA is widely distributed in the plant kingdom, and the main dietary sources include coffee beans and tea leaves, as well as many vegetables, such as potato, eggplant, carrot, and others [16,17]. The potential activity of CGA as a senotherapeutic agent has been demonstrated in several studies carried out both in vivo (gut aging, skin photoaging, and vascular senescence in mice) [17,18,19] and in vitro (senescent lens epithelial cells, fibroblasts, and keratinocytes) [20,21,22]. In particular, CGA and CGA-rich preparations modulate endothelial function and vascular aging mechanisms. In a preclinical study, CGA attenuated vascular senescence via the Nrf2/HO-1 axis, with concomitant effects on Sirt1 and eNOS [19], whereas human trials with CGA-rich extracts report time-dependent improvements in flow-mediated dilation alongside the biphasic appearance of CGA metabolites in plasma [23,24]. All these studies support an antioxidative mechanism involved in the senotherapeutic effects of CGA.

Given the lack of existing data on adipose tissue senescence, the aim of the present study was to investigate the protective effects of CGA against oxidative stress-induced senescence in adipocytes. In particular, we used an in vitro model of murine preadipocytes exposed intermittently to hydrogen peroxide (H_2_O_2_). This experimental setup provides a reproducible and controlled system to mimic senescence-associated dysfunction in adipose tissue and represents a valuable tool for the preclinical evaluation of natural compounds with potential therapeutic relevance [25,26]. Using this model, we assessed whether treatment with CGA during the differentiation stage could prevent senescence-related alterations and functional impairments, supporting its potential role in preserving adipose tissue integrity under stress conditions.

## 2. Results

### 2.1. Chlorogenic Acid Reduces Cellular Senescence in Adipocytes

Adipose tissue has long been considered an energy storage and endocrine organ. However, adipose tissue aging, associated with the accumulation of senescent cells within adipose tissue, leads to a progressive decline in tissue functionality, with significant implications for overall health and longevity.

**Figure 1:**
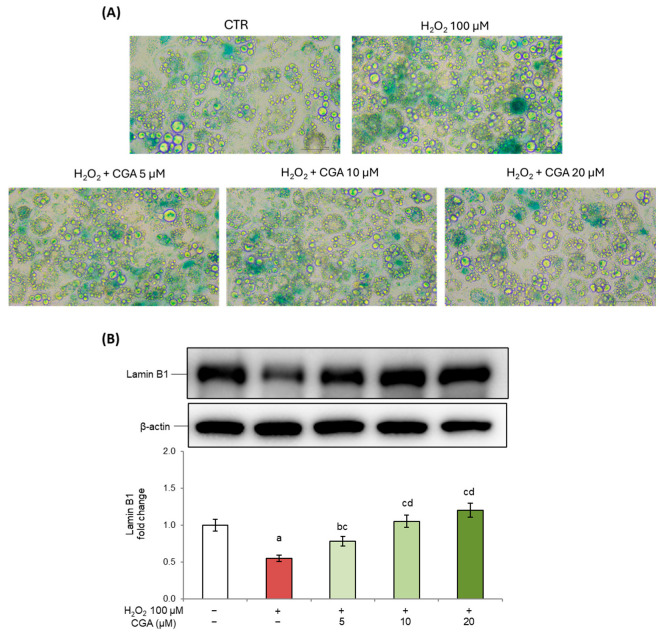
SA-β-Gal activity and Lamin B1 expression. Here, 3T3-L1 adipocytes were treated with H_2_O_2_ (100 μM) on days 5, 6, and 7 of the differentiation process for three hours, followed by CGA exposure (5, 10, or 20 μM), which was maintained until the end of the experiment (day 10). Cells cultured with the differentiation medium containing the CGA vehicle alone (0.1% *v*/*v* DMSO) were used as controls. (**A**) Representative images of SA-β-Gal staining (40× magnification—scale bar 50 µm) in control cells (CTR) and in cells exposed to H_2_O_2_ and treated or not with CGA. (**B**) Lamin B1 protein expression was analyzed by Western blot. The densitometry results are reported as fold changes compared to control cells. The values were normalized to the corresponding β-actin value. All results are reported as the mean ± S.D. of three independent experiments (n = 3 biological replicates). Error bars represent S.D. ^a^ *p* < 0.01 vs. control cells; ^b^ *p* < 0.05 vs. control cells; ^c^ *p* < 0.01 vs. H_2_O_2_; ^d^ *p* < 0.01 vs. H_2_O_2_ + CGA 5 μM.

In our experimental model, to investigate the induction of cellular senescence and to assess the potential protective effects of CGA, we evaluated two widely validated adipocyte markers of senescence: SA-β-Gal activity and Lamin B1 expression [27,28,29]. Our results revealed that, following exposure to H_2_O_2_, 3T3-L1 adipocytes displayed a marked increase in SA-β-Gal staining. Interestingly, CGA treatment significantly reduced SA-β-Gal staining in a dose-dependent way. In particular, at the highest tested concentration (20 µM), β-galactosidase activity was comparable to that observed in untreated controls, thereby supporting the protective effects of CGA against senescence in murine adipocytes (Figure 1A).

At the same time, we assessed the expression of Lamin B1, a key component of the nuclear envelope, whose downregulation reflects chromatin remodeling and transcriptional changes. Lamin B1 loss is recognized as a marker of senescence in response to all canonical signals of senescence [30]. In our experimental conditions, the data showed the marked downregulation of Lamin B1 levels following exposure to H_2_O_2_ compared to untreated controls, further confirming the establishment of a senescent state. Notably, CGA treatment dose-dependently restored its expression starting from 5 µM, suggesting a protective role in maintaining nuclear integrity (Figure 1B). For both parameters examined, no significant changes were found in 3T3-L1 cells exposed to CGA alone compared to control cells (see Figure S1 of Supplementary Materials).

### 2.2. Effects of Chlorogenic Acid on Cell Cycle Checkpoint Pathways

It is well established that one of the features of senescent cells is the upregulation of cyclin-dependent kinase inhibitors (CDKIs), which determine irreversible cell cycle arrest [31]. In particular, the p53/p21 axis plays a pivotal role since, upon DNA damage, the tumor suppressor protein p53 acts as a transcription factor for several stress-responsive genes, including Cdkn1a, which encodes for the CDK inhibitor p21. Elevated p21 expression levels lead, in fact, to G1/S arrest, considered a hallmark for the onset and maintenance of the senescent phenotype [32,33]. Since p53 activation is regulated by post-translational modifications regulating its stability and transcriptional activity, the phosphorylated form (Phospho-p53) was analyzed as a specific marker of its functional activation under oxidative stress conditions. In contrast, p21 expression mainly reflects transcriptional induction downstream of activated p53.

**Figure 2:**
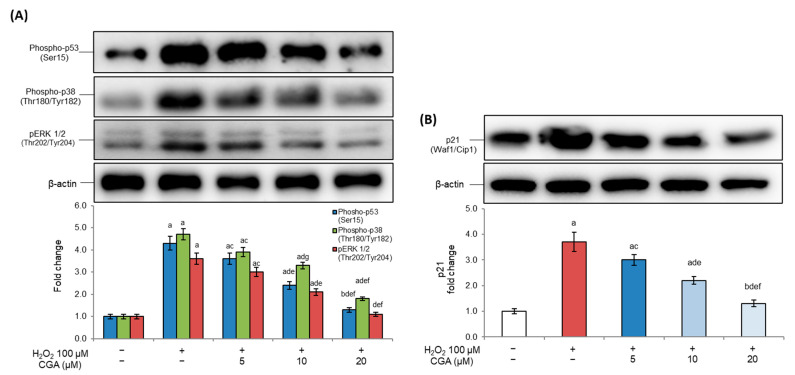
Effects of chlorogenic acid on cell cycle checkpoint pathways. Here, 3T3-L1 adipocytes were treated with H_2_O_2_ (100 μM) on days 5, 6, and 7 of the differentiation process for three hours, followed by CGA exposure (5, 10, or 20 μM). Cells cultured with the differentiation medium containing the CGA vehicle alone (0.1% *v*/*v* DMSO) were used as controls. (**A**,**B**) Phospho-p53, p21, phosphorylated p38, and pERK1/2 protein expression was analyzed by Western blot. The densitometry results are reported as fold changes compared to control cells. The values were normalized to the corresponding β-actin value. All results are reported as the mean ± S.D. of three independent experiments (n = 3 biological replicates). Error bars represent S.D. ^a^ *p* < 0.01 vs. control cells; ^b^ *p* < 0.05 vs. control cells; ^c^ *p* < 0.05 vs. H_2_O_2_; ^d^ *p* < 0.01 vs. H_2_O_2_; ^e^ *p* < 0.01 vs. H_2_O_2_ + CGA 5 μM; ^f^ *p* < 0.01 vs. H_2_O_2_ + CGA 10 μM; ^g^ *p* < 0.05 vs. H_2_O_2_ + CGA 5 μM.

Based on these considerations, to further explore the mechanisms underlying CGA’s protective effects, we investigated its impacts on key regulators of the cell cycle checkpoint pathway associated with senescence. Our data demonstrated that H_2_O_2_ exposure led to a marked increase in the levels of Phospho-p53 (Figure 2A), along with its downstream effector p21 (Figure 2B), confirming the onset of senescence-induced growth arrest.

Interestingly, CGA treatment attenuated the activation of these senescence-related pathways in a dose-dependent way. Even at the lowest tested concentration, CGA was, in fact, able to significantly reduce Phospho-p53 and p21 expression.

Additionally, mitogen-activated protein kinase (MAPK) family members, particularly p38 MAPK and ERK1/2 (pERK, phosphorylated extracellular signal-regulated kinases 1/2), further contribute to senescence establishment by inhibiting cell proliferation and differentiation and by promoting the activation of SASP [34]. In fact, the growth arrest of senescent cells modulated by CDKIs is under the direct control of MAPKs, particularly ERK1/2 and p38 [34]. In our experimental conditions, elevated levels of phosphorylated p38 (Phospho-p38; Figure 2A) and pERK1/2 (Figure 2A) were detected in H_2_O_2_-exposed adipocytes, underscoring their involvement in cell cycle arrest. Moreover, in this case, CGA treatment was able to reduce Ph-p38 and pERK1/2 levels in a dose-dependent way, starting from 5 µM. CGA treatment alone did not alter the analyzed parameters with respect to control cells (see Figure S2 of Supplementary Materials). These findings therefore suggest that CGA may alleviate senescence by modulating upstream sensors and downstream effectors involved in checkpoint regulation.

### 2.3. Effects of Chlorogenic Acid on Cell Proliferation and Apoptotic Regulation

**Figure 3:**
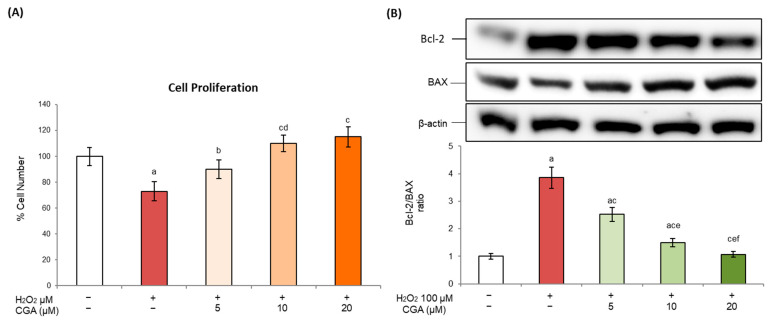
Effects of chlorogenic acid on apoptotic regulation. Here, 3T3-L1 adipocytes were treated with H_2_O_2_ (100 μM) on days 5, 6, and 7 of the differentiation process for three hours, followed by CGA exposure (5, 10, or 20 μM). Cells cultured with the differentiation medium containing the CGA vehicle alone (0.1% *v*/*v* DMSO) were used as controls. (**A**) Cell proliferation was determined by hemocytometer counts, and results are expressed as percentages relative to controls. (**B**) Bcl-2 and BAX proteins were analyzed by Western blot. The densitometry results, normalized to the corresponding β-actin value, were used to evaluate the Bcl-2/BAX ratio. All results are reported as the mean ± S.D. of three independent experiments (n = 3 biological replicates). Error bars represent S.D. ^a^ *p* < 0.01 vs. control cells; ^b^ *p* < 0.05 vs. H_2_O_2_; ^c^ *p* < 0.01 vs. H_2_O_2_; ^d^ *p* < 0.05 vs. H_2_O_2_ + CGA 5 μM; ^e^ *p* < 0.01 vs. H_2_O_2_ + CGA 5 μM; ^f^ *p* < 0.05 vs. H_2_O_2_ + CGA 10 μM.

Cellular senescence is characterized by cell proliferation arrest and resistance to apoptosis [35]. The inability of senescent cells to re-enter the cell cycle is indeed a key feature of this state, resulting in reduced tissue regeneration and homeostasis and in the formation of dysfunctional cells in aged or stressed tissues [36]. Our data confirmed that, in our experimental model, H_2_O_2_ significantly reduced the number of mature 3T3-L1 adipocytes (Figure 3A) and altered the expression of key apoptotic regulators. More specifically, H_2_O_2_ exposure resulted in elevated levels of the anti-apoptotic protein Bcl-2 (Figure 3B), accompanied by the marked downregulation of the pro-apoptotic protein BAX (Figure 3B). This shift toward an enhanced anti-apoptotic profile supports the survival of senescent cells and reflects a cellular environment favoring resistance to apoptosis. Notably, CGA treatment was able to reverse these alterations in a dose-dependent way. In fact, CGA treatment restored the proliferative capacity, as indicated by the increased cell number, and modulated the Bcl-2/BAX ratio to values comparable to those observed in controls. Meanwhile, CGA alone did not induce any detectable changes compared to control cells (see Figure S3 of Supplementary Materials). These findings underscore the ability of CGA to counteract impaired cellular proliferation and resistance to apoptosis, thereby reinforcing its therapeutic potential as a senomorphic agent in preserving adipose tissue homeostasis.

### 2.4. Chlorogenic Acid’s Effects in Counteracting SASP Oxidative and Inflammatory Response

**Figure 4:**
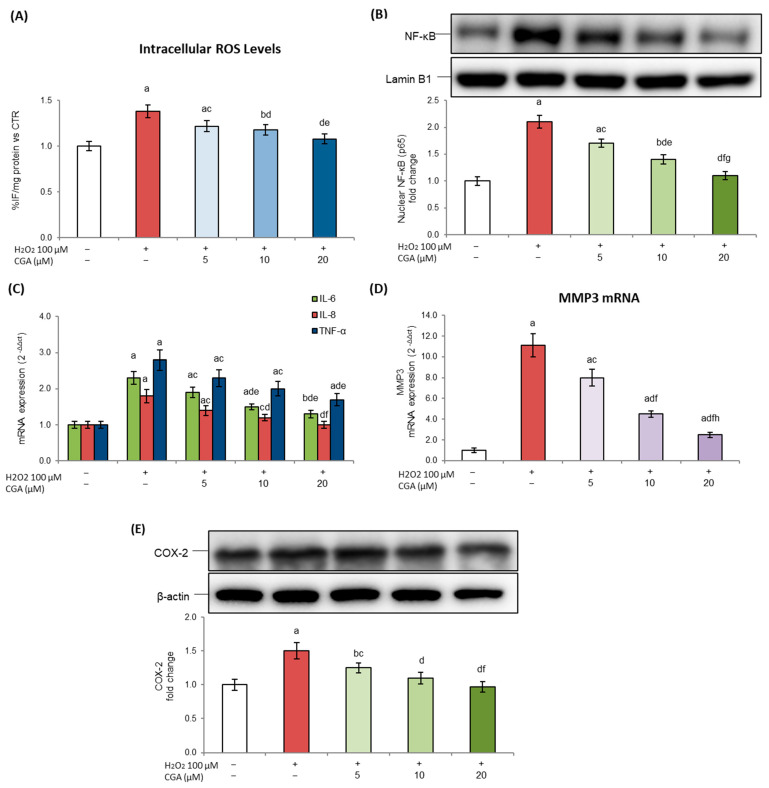
Effects of chlorogenic acid on oxidative stress, inflammatory signaling, and ECM remodeling factors. Here, 3T3-L1 adipocytes were treated with H_2_O_2_ (100 μM) on days 5, 6, and 7 of the differentiation process for three hours, followed by CGA exposure (5, 10, or 20 μM). Cells cultured with the differentiation medium containing the CGA vehicle alone (0.1% *v*/*v* DMSO) were used as controls. (**A**) Intracellular ROS levels are reported as the % change in fluorescence intensity/mg of proteins against controls. (**B**) Nuclear NF-κB (p65) protein expression was analyzed by Western blot. The densitometry results are reported as fold changes compared to control cells; values were normalized to the corresponding Lamin B1 value. (**C**,**D**) IL-6, IL-8, TNF-α, and MMP-3 gene expression values are expressed as 2^−ΔΔCt^ and normalized against control cells. 18S rRNA was used as a housekeeping gene. (**E**) COX-2 protein expression was analyzed by Western blot. The densitometry results are reported as fold changes compared to control cells; values were normalized to the corresponding β-actin value. All results are reported as the mean ± S.D. of three independent experiments (n = 3 biological replicates). Error bars represent S.D. ^a^ *p* < 0.01 vs. control cells; ^b^ *p* < 0.05 vs. control cells; ^c^ *p* < 0.05 vs. H_2_O_2_; ^d^ *p* < 0.01 vs. H_2_O_2_; ^e^ *p* < 0.05 vs. H_2_O_2_ + CGA 5 μM; ^f^ *p* < 0.01 vs. H_2_O_2_ + CGA 5 μM; ^g^ *p* < 0.05 vs. H_2_O_2_ + CGA 10 μM; ^h^ *p* < 0.01 vs. H_2_O_2_ + CGA 10 μM.

An important feature of SASP is the accumulation of ROS [37], which not only trigger oxidative damage but also act as activators of the inflammatory pathways defining the SASP profile. In our experimental model, H_2_O_2_ exposure significantly increased the intracellular ROS levels, indicating the onset of an oxidative stress state. Interestingly, CGA treatment was able to reduce them in a dose-dependent way, with values, for the highest tested concentration, that were even lower than those observed in control cells (Figure 4A).

Given the central role of ROS in promoting inflammatory signaling, we next assessed the expression of NF-κB, the master regulator of inflammation activated in response to oxidative stress. In fact, NF-κB acts as a key modulator able to induce and sustain the expression of multiple SASP factors, including pro-inflammatory cytokines and matrix remodeling enzymes [36]. The results obtained showed that H_2_O_2_ treatment markedly increased NF-κB expression, whereas CGA significantly reduced its levels in a dose-dependent way (Figure 4B).

Since NF-κB is a key modulator of the inflammatory SASP response, we then examined its principal downstream effectors.

It is widely known that SASP activation leads to an increase in the release of pro-inflammatory cytokines and matrix remodeling enzymes, which contribute to tissue dysfunction and chronic inflammation [38]. In this context, in our study, to further investigate the potential activity of CGA to modulate the senescence phenotype, we assessed the expression levels of the pro-inflammatory cytokines interleukin-6 (IL-6), interleukin-8 (IL-8), and tumor necrosis factor-α (TNF-α), which are well-established SASP components and important contributors to adipose tissue dysfunction. These cytokines play a central role in the onset of the low-grade chronic inflammatory state typical of senescent adipose tissue, where they promote metabolic dysregulation, immune cell infiltration, and extracellular matrix (ECM) remodeling [36]. IL-6 and TNF-α, in particular, are closely linked to impaired insulin signaling and adipocyte dysfunction. Meanwhile, IL-8 has an important role as a chemoattractant for immune cells and as a promoter of senescence propagation [39,40]. As expected, H_2_O_2_ exposure induced the strong upregulation of all three cytokines, confirming the establishment of a pro-inflammatory SASP phenotype. Notably, CGA treatment reduced IL-6, IL-8, and TNF-α expression in a dose-dependent way, significantly counteracting the inflammatory response triggered by oxidative stress (Figure 4C). To further characterize the SASP profile, we also examined the expression of matrix metalloproteinase 3 (MMP3), an important enzyme modulated by NF-κB, involved in the degradation of ECM components and considered not only a structural modulator but also a key SASP effector that amplifies inflammatory signaling and contributes to tissue remodeling in aging- and senescence-related pathologies [41,42].

Our data demonstrated that H_2_O_2_ exposure resulted in the marked upregulation of MMP-3 (Figure 4D) gene expression, reinforcing the onset of a SASP profile in senescent 3T3-L1 adipocytes. Interestingly, in this case, CGA treatment was also able to reduce its expression starting from 5 µM.

Furthermore, we analyzed the expression of cyclooxygenase-2 (COX-2), an inducible enzyme involved in the synthesis of pro-inflammatory prostaglandins, commonly induced under oxidative and inflammatory conditions and contributing to the chronic inflammatory state associated with cellular senescence [43]. As shown in Figure 4E, we observed the marked upregulation of COX-2 following treatment with H_2_O_2_. Notably, CGA treatment downregulated COX-2 expression in a dose-dependent manner.

Moreover, in this case, CGA exposure alone did not induce any detectable changes in ROS, NF-κB, and COX-2 levels or in IL-6, TNF-α, IL-8, and MMP-3 gene expression compared to control cells (see Figure S4 of Supplementary Materials).

Altogether, these findings support CGA’s ability to modulate oxidative stress and reduce the inflammatory and matrix remodeling components of SASP.

### 2.5. Effects of Chlorogenic Acid on the Insulin Signaling Pathway in Senescent Adipocytes

In adipose tissue, cellular senescence is not only associated with chronic inflammation and structural remodeling but also with the onset of several types of metabolic dysfunction [44]. In particular, one of the main consequences of senescence-related dysfunction in adipose tissue is the impairment of insulin signaling, which reduces glucose homeostasis, contributing to the development of an insulin resistance state [11]. In senescent adipocytes, oxidative stress interferes with insulin signaling by suppressing the phosphatidylinositol 3-kinase (PI3K)–AKT pathway, an important regulator of insulin-mediated metabolic responses [45]. This downregulation leads to the reduced expression and translocation of glucose transporter 4 (GLUT4), compromising glucose uptake and promoting systemic metabolic dysregulation [46].

**Figure 5:**
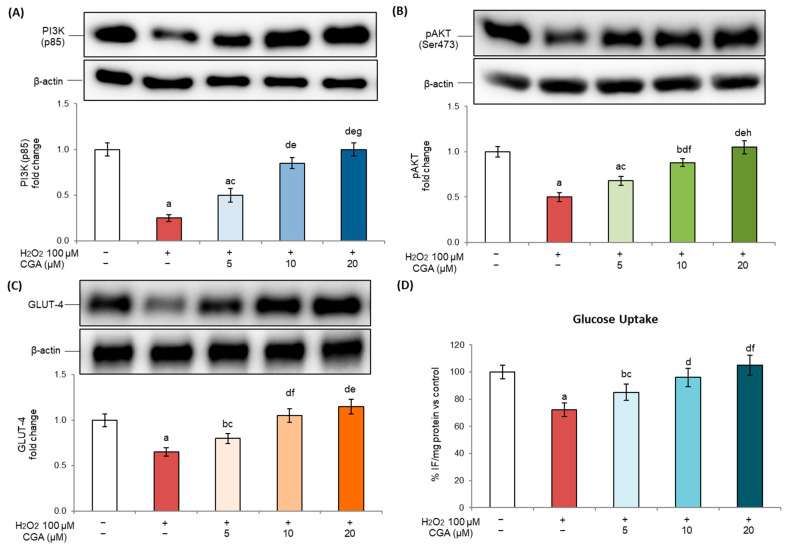
Effects of chlorogenic acid on insulin signaling pathway. Here, 3T3-L1 adipocytes were treated with H_2_O_2_ (100 μM) on days 5, 6, and 7 of the differentiation process for three hours, followed by CGA exposure (5, 10, or 20 μM). Cells cultured with the differentiation medium containing the CGA vehicle alone (0.1% *v*/*v* DMSO) were used as controls. (**A**–**C**) The expression levels of PI3K, pAKT, and GLUT-4 proteins were analyzed by Western blot. The densitometry results are reported as fold changes compared to control cells. The values were normalized to the corresponding β-actin value. (**D**) Glucose uptake results are reported as the % change in fluorescence intensity/mg of proteins against controls. All results are reported as the mean ± S.D. of three independent experiments (n = 3 biological replicates). Error bars represent S.D. ^a^ *p* < 0.01 vs. control cells; ^b^ *p* < 0.05 vs. control cells; ^c^ *p* < 0.05 vs. H_2_O_2_; ^d^ *p* < 0.01 vs. H_2_O_2_; ^e^ *p* < 0.01 vs. H_2_O_2_ + CGA 5 μM; ^f^ *p* < 0.05 vs. H_2_O_2_ + CGA 5 μM; ^g^ *p* < 0.05 vs. H_2_O_2_ + CGA 10 μM; ^h^ *p* < 0.01 vs. H_2_O_2_ + CGA 10 μM.

Our results showed that exposure to H_2_O_2_ significantly inhibited PI3K activation (Figure 5A) with consequent reduced AKT phosphorylation (Figure 5B) and GLUT4 expression (Figure 5C), confirming the onset of an insulin-resistant state in senescent 3T3-L1 adipocytes. At the same time, glucose uptake (Figure 5D) was also markedly reduced, providing further evidence of impaired insulin signaling and highlighting the compromised metabolic functionality associated with cellular senescence. Interestingly, CGA treatment was able to counteract these alterations in a dose-dependent way. CGA, in fact, restored PI3K-AKT pathway activation and enhanced both GLUT4 expression levels and glucose uptake, suggesting an improvement in insulin responsiveness in senescent adipocytes. No changes were observed in cells treated with CGA alone compared to controls (see Figure S5 of Supplementary Materials). These results therefore suggest an important role of CGA in preventing insulin resistance associated with cellular senescence and aging.

### 2.6. Chlorogenic Acid’s Effects on Adipogenesis and Lipid Accumulation in Senescent 3T3-L1 Cells

One of the main consequences of cellular senescence is the disruption of adipogenic potential due to the alteration of functional markers involved in adipocyte differentiation [47]. In physiological conditions, in fact, the adipogenesis process is a well-orchestrated program that—through the sequential activation of early and late transcription factors, including peroxisome proliferator-activated receptor gamma (PPARγ)—plays a key role in the full differentiation of the mature adipocyte. Furthermore, the expression of PPARγ-regulated specific genes including fatty acid synthase (FASN) and fatty acid binding protein 4 (FABP4) further contributes to adipose tissue development by promoting lipid biosynthesis and accumulation [48,49].

**Figure 6:**
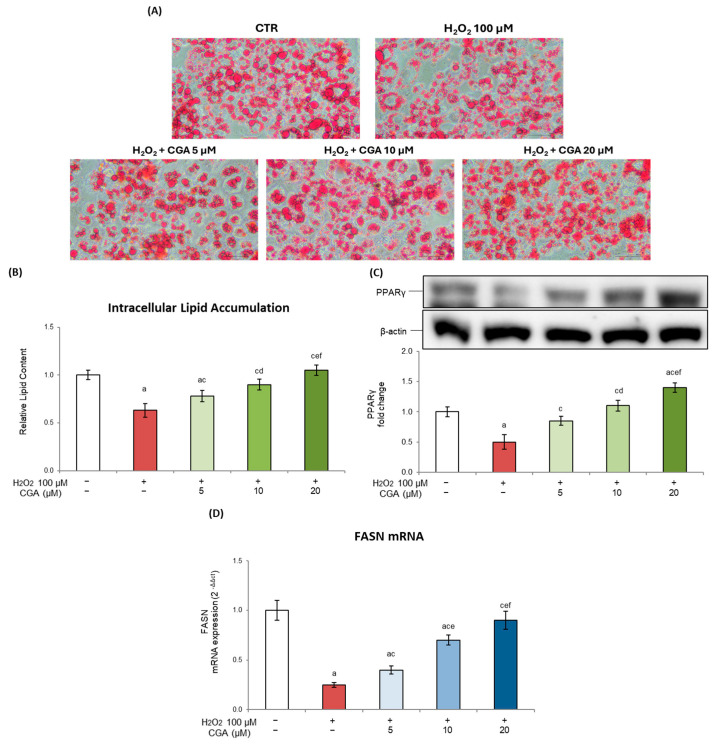
Effects of chlorogenic acid on adipogenesis. Here, 3T3-L1 adipocytes were treated with H_2_O_2_ (100 μM) on days 5, 6, and 7 of the differentiation process for three hours, followed by CGA exposure (5, 10, or 20 μM). Cells cultured with the differentiation medium containing the CGA vehicle alone (0.1% *v*/*v* DMSO) were used as controls. (**A**) Representative images of Oil Red O staining (original magnification at ×40—scale bar 50 µm) in control cells (CTR) and in cells exposed to H_2_O_2_ and treated or not with CGA. (**B**) Cell lipid accumulation was expressed as relative content vs. control cells. (**C**) PPARγ protein expression was analyzed by Western blot. The densitometry results are reported as fold changes compared to control cells. The values were normalized to the corresponding β-actin value. (**D**) FASN gene expression value was expressed as 2^−ΔΔCt^ and normalized against control cells. 18S rRNA was used as a housekeeping gene. All results are reported as the mean ± S.D. of three independent experiments (n = 3 biological replicates). Error bars represent S.D. ^a^ *p* < 0.01 vs. control cells; ^c^ *p* < 0.01 vs. H_2_O_2_; ^d^ *p* < 0.05 vs. H_2_O_2_ + CGA 5 μM; ^e^ *p* < 0.01 vs. H_2_O_2_ + CGA 5 μM; ^f^ *p* < 0.05 vs. H_2_O_2_ + CGA 10 μM.

In the presence of cellular senescence, an impaired differentiation capacity and dysfunctional lipid metabolism occur, which determine several alterations to the adipogenic program, thus contributing to metabolic decline in aging adipose tissue [33,50]. In our experimental model, H_2_O_2_ significantly impaired adipogenesis, as shown by Oil Red O staining, which revealed a marked reduction in intracellular lipid droplets compared to untreated control cells (0.63 ± 0.11 fold vs. CTR, *p* < 0.05) (Figure 6A,B). This effect was associated with the strong downregulation of PPARγ levels (Figure 6C) following H_2_O_2_ exposure, indicating an altered differentiation program. The transcriptional regulation of PPARγ was further supported by FASN gene expression (Figure 6D), which was significantly reduced by H_2_O_2_, thereby confirming the lipogenic function impairment in senescent cells. Remarkably, CGA treatment effectively counteracted these effects in a dose-dependent way. CGA treatment, in fact, enhanced lipid accumulation, as demonstrated by an increase in both the size and number of lipid droplets compared to control cells (0.78 ± 0.12, 0.93 ± 0.10, and 1.03 ± 0.08 for CGA at 5, 10, and 20 μM, respectively). At the same time, CGA restored both PPARγ protein expression (Figure 6C) and FASN mRNA levels (Figure 6D), suggesting that CGA not only preserves the differentiation capacity but also supports functional lipid metabolism in senescent adipocytes. Notably, also in this case, no significant changes were found in 3T3-L1 cells exposed to CGA alone compared to control cells (see Figure S6 of Supplementary Materials).

## 3. Discussion

Cellular senescence represents a permanent state of cell cycle arrest, with broad implications for aging, cancer, and chronic disorders. Initially described as a tumor-suppressive mechanism preventing the expansion of damaged cells, senescence is nowadays considered a complex event that promotes tissue dysfunction and plays a pivotal role in the onset of age-related pathologies [51]. Although the accumulation of senescent cells has been extensively described in aging and in several chronic diseases, including obesity and diabetes [52,53], little is currently known about strategies that are able to mitigate or reprogram senescence in adipose tissue.

In this study, we aimed to investigate the protective effects of CGA against adipocyte senescence, supporting its potential role as a senotherapeutic agent in adipose tissue. CGA is a naturally occurring polyphenolic compound, abundantly present in many plant sources, including green coffee beans, tea, and several fruits and vegetables. CGA exerts different therapeutic effects in response to a variety of pathological conditions, particularly those associated with chronic metabolic diseases and age-related disorders [54]. Importantly, the senotherapeutic activity of CGA has already been reported in other tissues, such as the gut, skin, and vasculature, where its effects have been primarily attributed to antioxidant and anti-inflammatory mechanisms [15,19]. The results of the present work therefore serve to expand this knowledge, showing that CGA exerts protective actions against adipocyte senescence, including redox homeostasis, inflammatory signaling, insulin responsiveness, and adipogenic processes. These effects may contribute to resilience pathways that support endothelial stability and mitochondrial health, as discussed in the context of redox-driven vascular and metabolic aging [55,56].

Senescent cells display a wide range of structural and biochemical alterations, such as increased and flattened cell sizes, associated with the expression of SA-β-Gal [9]. We have demonstrated that CGA attenuates senescence markers in H_2_O_2_-induced senescence in adipocytes, as evidenced by the reduction in SA-β-Gal activity (Figure 1A) and the restoration of Lamin B1 expression (Figure 1B), another well-established hallmark of cellular senescence [47,57,58]. These effects may be explained by CGA’s ability to attenuate oxidative stress and modulate stress response pathways. Notably, p53/p21 signaling and MAPK activation are key drivers of Lamin B1 loss and lysosomal hyperactivity during senescence [57,59]. Since Lamin B1 is a structural component of the nuclear envelope and a key target of chromatin reorganization, these findings suggest that CGA may stabilize nuclear integrity, counteracting the chromatin remodeling and nuclear alterations typical of senescent cells.

In line with these findings, and considering that senescent cells are characterized by genomic instability that activates DNA damage responses following stimulation with intrinsic and extrinsic stressors [60], we further explored the modulation of cell cycle checkpoint pathways. In particular, the senescent response is especially regulated by cyclin-dependent kinase inhibitors, such as p16INK4a and the p53/p21 pathway, which enforce growth arrest by interfering with essential regulators of the cell cycle [36]. H_2_O_2_-induced senescent adipocytes showed the upregulation of phosphorylated p53 and its downstream effector p21 (Figure 2A,B), confirming the establishment of cellular growth arrest [61]. CGA treatment, instead, significantly reduced Phospo-p53 levels, whose inactivation can disrupt the onset of the senescence process [62], as well as p21 expression, indicating CGA’s ability to modulate stress-induced checkpoint activation and restore a proliferative state in 3T3-L1 adipocytes. These observations are in accordance with previous studies that showed the capacity of CGA and other dietary polyphenols to modulate p53/p21 signaling, thereby preventing oxidative damage and delaying senescence development [9,63,64]. The DNA damage response represents a central signaling cascade that not only triggers and sustains cellular senescence but arises in connection with mechanisms linked to oxidative stress, mitochondrial function, and vascular resilience. Recent evidence highlights that redox-regulated pathways contribute to endothelial and mitochondrial adaptation during aging, influencing both metabolic and vascular homeostasis [65]. In this context, stress-activated kinases of the MAPK family, particularly p38 and pERK1/2, play a key role in translating oxidative and genotoxic stress signals into a senescent phenotype. The activation of p38 MAPK, in fact, is closely related to the stabilization of p53 and the induction of its downstream effector p21, thereby reinforcing growth arrest and promoting SASP establishment [66]. At the same time, pERK1/2 activation has been associated with premature senescence, promoting both growth arrest and SASP-related gene expression [67]. In our model, CGA treatment attenuated p38 phosphorylation and pERK1/2 activity, suggesting that this compound interferes with stress-induced MAPK signaling cascades. This effect, combined with the downregulation of Phospo-p53 and p21, indicates CGA’s capacity to reprogram the cell cycle at multiple levels. In fact, our data confirmed that, in this model of adipocyte senescence, CGA treatment restored the cell number decreased by exposure to H_2_O_2_.

One important feature of senescent cells is that they become resistant to apoptosis by activating specific antiapoptotic cellular pathways. It has been demonstrated that both senescence and apoptosis trigger the p53 pathway, whose activation is crucial for senescent cells to resist apoptosis and to enter permanent cell cycle arrest [68]. Furthermore, the activation of p38 and pERK1/2 signaling may further reinforce the anti-apoptotic phenotype through the modulation of transcriptional programs and survival pathways that limit cell death [69]. In our experimental conditions, H_2_O_2_ exposure produced a marked anti-apoptotic shift, resulting in a Bcl-2 increase and BAX downregulation (Figure 3). Interestingly, CGA treatment reversed all these effects in a dose-dependent way, suggesting the restoration of apoptotic mechanisms in senescent adipocytes. These data are in accordance with those obtained in other experimental models and showing that CGA, through antioxidant and signaling-modulatory properties, contributes to regulating cell proliferation and apoptosis, thereby reprogramming stressed cells toward a more homeostatic condition [70,71]. However, it should be noted that the restoration of the proliferative capacity and apoptotic mechanisms can have dual consequences. It is well known that, at the oncological level, senescence acts as a tumor suppression mechanism, preventing damaged cells from re-entering the cell cycle; consequently, all therapeutic strategies aimed at reactivating the cell cycle must be carefully evaluated in order to prevent an unintentional reduction in this protective barrier [72].

In this regard, several studies have shown how cells with DNA damage can alter senescence and excessive cell proliferation, resulting in genomic instability and a higher risk of malignant transformation [73,74,75]. Likewise, the excessive elimination of senescent cells through the induction of apoptotic mechanisms may alter the tissue architecture or trigger the compensatory proliferation of neighboring cells, leading to tumor promotion [76,77]. However, it should be underlined that our results were obtained in mature adipocytes, a terminally differentiated and non-proliferative cell type with low tumorigenic potential. In this context, the increase in cell number observed after CGA treatment reflects restored cell viability rather than cell cycle re-entry. Taken together, these observations therefore support CGA’s role as a senomorphic compound, restoring adipocyte homeostasis without promoting pathological proliferation or compromising the tumor-suppressive aspects of the senescence program.

Senescent cells are characterized by the increased secretion of cytokines, chemokines, proteases, and growth factors, which act via autocrine and paracrine pathways to enhance and diffuse senescent cells’ influence and induce chronic inflammation in adipose tissue [11]. This leads to the establishment of systemic low-grade inflammation, contributing to tissue dysfunction [78]. A central driver of this secretory program is the transcription factor NF-κB, which is activated under oxidative and inflammatory conditions and orchestrates the expression of key SASP components, including IL-6, IL-8, TNF-α, and matrix remodeling enzymes. In our experimental model, H_2_O_2_ exposure markedly increased the intracellular ROS levels, consistent with the oxidative stress-mediated activation of NF-κB.

According to this, our data confirmed SASP activation in H_2_O_2_-exposed adipocytes, as shown by higher IL-6, IL-8, TNF-α, and MMP-3 mRNA levels. Similarly, senescent adipocytes showed increased COX-2 expression, further supporting the establishment of an inflammatory phenotype. In addition, the increase in ROS levels confirms the involvement of oxidative stress in H_2_O_2_-induced senescence and supports its role as an upstream trigger of NF-κB activation, which in turn drives the pro-inflammatory SASP response. In our experiments, CGA treatment effectively attenuated these alterations, reducing ROS accumulation, downregulating NF-κB expression, and consequently decreasing the expression of SASP-related pro-inflammatory cytokines and matrix remodeling factors induced by H_2_O_2_ (Figure 4). These data are in accordance with a previous report demonstrating CGA’s ability to reduce SASP factors and inhibit the elevation in ROS levels in human dermal skin fibroblasts exposed to UVA [79]. By counteracting inflammatory cytokine release and matrix remodeling enzymes, CGA therefore acts on SASP components, mitigating the pro-inflammatory and tissue-disrupting features of senescent adipocytes.

The aging of adipose tissue has a significant impact on insulin sensitivity, which may be followed by the development of diabetic conditions [80]. In addition, during senescence, the increase in inflammatory mediators can interfere with insulin signaling, thereby contributing to the development of insulin resistance [44]. Our results are consistent with these observations, since H_2_O_2_-induced senescence reduced insulin sensitivity and glucose uptake. We further demonstrated that CGA treatment was able to restore the insulin pathway and recover glucose uptake, which was altered following treatment with H_2_O_2_ (Figure 5). Although previous studies based on in vitro and in vivo models have shown that CGA, as well as other polyphenols, is able to improve insulin sensitivity and glucose uptake [81,82,83], this is the first time that CGA has been demonstrated to be able to protect against insulin resistance in cellular senescence.

In addition to impaired insulin sensitivity, the onset of senescence can interfere with adipocyte differentiation and lipid metabolism. Senescent preadipocytes, in fact, have a reduced capacity to undergo adipogenesis, resulting in impaired lipid accumulation, which contributes to adipose tissue dysfunction and systemic metabolic imbalance [11,84]. Furthermore, the onset of a SASP environment leads to alterations in key mediators of the process, further disrupting the transcriptional adipogenesis program. In agreement with these observations, under our experimental conditions, H_2_O_2_ exposure induced a dysfunctional state, impairing the differentiation potential of 3T3-L1 cells and altering lipid accumulation and the expression of key adipogenesis mediators. CGA treatment was able to counteract these effects, restoring a more physiological lipid storage pattern (Figure 6). It has been demonstrated that CGA can modulate adipogenesis and lipid metabolism in other experimental settings through multiple mechanisms, including the regulation of PPARγ signaling, the reduction of oxidative stress, and the modulation of AMPK activity [85,86,87]. These data therefore highlight CGA’s ability to promote a more balanced adipocyte phenotype under aging conditions.

## 4. Materials and Methods

### 4.1. Reagents

Dulbecco’s modified eagle medium (DMEM) and Dulbecco’s phosphate-buffered saline (DPBS) were purchased from Thermo Fisher Scientific (Monza, Italy). Hydrogen peroxide solution (3%—10 Vols) was acquired from Liofilchem (Teramo, Italy). Chlorogenic acid (CGA) and all other chemicals and solvents used in this study, unless otherwise indicated, were obtained from Merck Life Science (Milan, Italy).

### 4.2. Cell Culture and Treatments

Mouse 3T3-L1 preadipocytes (ATCC, Manassas, VA, USA) were cultured and differentiated into mature adipocytes according to the method described in our previous work [88]. Briefly, the cells were seeded at 1.3 × 10^4^ cells/cm^2^ in multiwell plates and, upon reaching confluence, were maintained in culture for 10 days, until complete differentiation into mature adipocytes. In detail, during the first 4 days (induction phase), cells were incubated in DMEM supplemented with 10% fetal bovine serum (FBS), 4 mM L-glutamine, 100 U/mL penicillin/streptomycin, and 25 mM HEPES buffer (DMEM-full), containing a mixture of 3 isobutyl-1-methylxanthine (0.5 mM), dexamethasone (Dex) 1 μM, and insulin (1 μg/mL) (MDI). Afterwards, this medium was replaced, and the cells were maintained, from day 4 to day 7, in DMEM-full containing only 1 μg/mL insulin. Finally, from day 7 to day 10, cells were cultured in insulin-free DMEM-full to obtain completely differentiated adipocytes.

To induce senescence, on days 5, 6, and 7 of the cell differentiation process, cells were exposed intermittently to a sub-lethal concentration of H_2_O_2_ (100 µM) for 3 h, following the method established by Zoico et al. [25]. In particular, on days 5 and 6, cells were treated for 3 h with H_2_O_2_; after this exposure period, cells were gently washed with DPBS, and insulin DMEM-full supplemented with different CGA concentrations was added until the next H_2_O_2_ exposure. On day 7, after the final H_2_O_2_ exposure, cells were gently washed with DPBS, and CGA was added in insulin-free DMEM-full and maintained until the end of the differentiation period (day 10). The CGA concentrations (5, 10, and 20 µM) were selected according to oral bioavailability studies [89] and consistent with physiologically relevant levels achievable through pharmacological or dietary intake [90].

**Figure 7:**
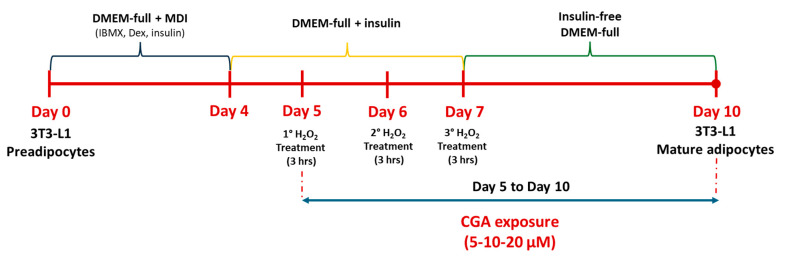
Schematic representation of the experimental model. Line colors indicate the culture media used during the differentiation process: blue = DMEM + MDI (IBMX, Dex, Ins), yellow = complete DMEM with insulin, green = complete DMEM without insulin.

Cells treated with the CGA vehicle alone (DMSO 0.1% *v*/*v*) and exposed only to the differentiation medium were used as controls. At the end of the treatment period (day 10), cells were processed immediately or stored at –80 °C until further analysis. A schematic representation of the experimental design is shown in Figure 7.

### 4.3. Senescence-Associated β-Galactosidase Staining

The senescence-associated β-galactosidase (SA-β-Gal) staining assay is a method that is widely used to identify senescent cells. In fact, increased activity of the β-galactosidase enzyme at pH 6 is a typical feature of senescent but not proliferating cells [91]. This test is based on the enzymatic hydrolysis of the chromogenic substrate 5-bromo-4-chloro-3-indolyl β-D-galactopyranoside (X-Gal), which produces an insoluble blue precipitate that accumulates in senescent cells and can be seen under an optical microscope. In particular, in this study, the SA-β-Gal staining procedure was performed following the protocol described by Itahana et al. [27], with minor modifications. In short, after the removal of the culture medium, cells were washed with DPBS and fixed with 4% formaldehyde for 5 min at room temperature. Subsequently, cells were washed again with DPBS and incubated with a freshly prepared SA-β-Gal staining solution at 37 °C for 12–16 h. At the end of the incubation period, the staining solution was removed, and the wells were rinsed with DPBS in order to eliminate the unbound dye. Blue-stained SA-β-Gal-positive cells were observed using an inverted microscope, and representative images were captured for further analyses.

### 4.4. Cell Proliferation

Cell proliferation was carried out using the Erythrosin B exclusion test. Erythrosin B solution (0.1% *w*/*v*) was prepared in DPBS, following the manufacturer’s instructions (Sigma-Aldrich, Milan, Italy). After trypsinization, cell counts were performed using a hemocytometer, and results were expressed as a percentage relative to untreated control cells.

### 4.5. Adipocyte Lipid Staining and Droplet Analysis

To evaluate intracellular lipid accumulation, adipogenic cultures were stained with Oil Red O (ORO), following the method described by Molonia et al. [92]. Subsequently, after fixation and staining, cells were visualized under an inverted microscope and photographed for further analysis. Quantification of lipid content was performed using the ImageJ software (v1.54g) [93]. Lastly, the Oil Red O stain retained in the cells was eluted with 100% isopropanol, and lipid accumulation was evaluated spectrophotometrically at 490 nm using a microplate reader (GloMax^®^ Discover System—TM397, Promega Corporation, Madison, Wisconsin, USA). Results were reported as fold changes relative to control cells.

### 4.6. Cell Lysate Preparation

Following the treatments, 3T3-L1 cells were washed with DPBS and harvested using a cell scraper. Total protein content was determined by incubating the samples for one hour at a low temperature in a lysis buffer (1 mM EDTANa_2_, 10 mM Tris–HCl, 150 mM NaCl, 0.1% SDS, 5% glycerol, and 1% Triton). Nuclear and cytoplasmatic extracts were prepared as previously described [94]. In more detail, the cells were firstly treated with a hypotonic buffer (10 mM HEPES, 10 mM KCl, 1.5 mM MgCl_2_, and 5% glycerol) to extract cytoplasmatic proteins and then incubated with a hypertonic buffer (20 mM HEPES, 1 mM MgCl_2_, 400 mM NaCl, 1 mM EGTA, 0.1 mM EDTA, and 10% glycerol) for the nuclear fractions. All lysis buffers contained protease inhibitors (1 μg/mL leupeptine, 2 μg/mL aprotinine, 1 mM benzamidine, and 5 mM NaF) and 1 mM dithiothreitol (DTT). Protein fractions were stored at −20 °C until further analysis, and protein concentrations were determined using the Bradford assay [95].

### 4.7. Immunoblotting

For immunoblot analyses, 30 µg of lysate was subjected to SDS-PAGE and then electrotransferred onto a PVDF membrane. Membranes were then blocked using 5% non-fat dry milk solution and incubated overnight at 4 °C with the following specific primary antibodies: rabbit anti-Lamin B1 monoclonal antibody (Cell Signaling Technology, Danvers, MA, USA) (1:1000), mouse anti-PPAR-γ monoclonal antibody (Santa Cruz Biotechnology, Dallas, TX, USA) (1:1500), rabbit anti-PI3K p85 monoclonal antibody (Cell Signaling Technology) (1:1000), mouse anti-p21 (Waf1/Cip1) monoclonal antibody (Santa Cruz Biotechnology) (1:500), mouse anti-phospho-p53 (Ser15) monoclonal antibody (Cell Signaling Technology) (1:1000), rabbit anti-phospho-p38 (Thr180/Tyr182) monoclonal antibody (Cell Signaling Technology) (1:1000), rabbit anti-phospho-ERK1/2 (Thr202/Tyr204) monoclonal antibody (Cell Signaling Technology) (1:1000), rabbit anti-Bax monoclonal antibody (Cell Signaling Technology) (1:1000), rabbit anti-Bcl-2 monoclonal antibody (Cell Signaling Technology) (1:1000), rabbit anti-Phospho-Akt (Ser473) monoclonal antibody (Cell Signaling Technology) (1:1000), rabbit anti-GLUT-4 monoclonal antibody (Cell Signaling Technology) (1:1000), rabbit anti-NF-κB p65 polyclonal antibody (Invitrogen, Milan, Italy) (1:1000), mouse anti-COX-2 monoclonal antibody (Santa Cruz Biotechnology) (1:500), and rabbit anti-β-actin monoclonal antibody (Cell Signaling Technology) (1:6000).

Subsequently, the membranes were incubated for 2 h at room temperature with peroxidase-conjugated secondary antibody HRP-labeled goat anti-rabbit Ig (Cell Signaling Technology) (1:6000) or goat anti-mouse IgM secondary antibody HRP conjugate (Cell Signaling Technology) (1:6000), and they were visualized using an ECL plus detection system (Amersham; GE Healthcare Life Sciences, Marlborough, MA, USA). Blots were detected using a ChemiDoc Imaging System (Bio-Rad, Hercules, CA, USA). The intensities of the bands were quantified using the Image Lab program, and the equivalent loading of proteins in each well was confirmed by Ponceau staining or β-actin.

### 4.8. Intracellular Reactive Oxygen Species

Intracellular reactive oxygen species (ROS) levels were determined using the fluorescent 2′,7′-dichlorodihydrofluorescein diacetate (DCFH-DA), following the protocol described by Bashllari et al. [96]. After treatment, cells were rinsed twice with DPBS and then incubated with 50 μM DCFH-DA at 37 °C for 30 min. The fluorescence intensity was measured using a microplate reader (GloMax^®^ Discover System—TM397) (excitation 485 nm and emission 530 nm). The assay was performed in triplicate. ROS levels were normalized to the total protein content and expressed as the DCFH-DA relative fluorescence intensity per mg of protein against controls.

### 4.9. Real-Time PCR

Total RNA was extracted using the E.Z.N.A.^®^ Total RNA kit, following the manufacturer’s instructions (OMEGA bio-tek, Norcross, GA, USA); quantified by a Quant-iT TM RNA assay kit (Invitrogen, Milan, Italy); and reverse-transcribed with M-MLV Reverse Transcriptase. A quantitative real-time polymerase chain reaction (PCR; Applied Bio systems 7300 Real-Time PCR System, Thermo Fisher, Milan, Italy) coupled with SYBR green chemistry (SYBR green JumpStart Taq Ready Mix, Sigma-Aldrich, Milan, Italy) was carried out for the identification of the mRNA levels of FASN (FW 5′-GGAGGTGGTGATAGCCGGTAT-3′, RV 5′-TGGGTAATCCATAGAGCCCAG-3′ [97]), IL-8 (FW 5′-GCACTTGGGAAGTTAACGCA-3′, RV 5′-GCACAGTGTCCCTATAGCCC-3′ [98]), MMP-3 (FW 5′-TGATGAACGATGGACAGAGG-3′, RV 5′-GAGAGATGGAAACGGGACAA-3′ [99,100]), and p21 (FW 5′-CTAGGGGAATTGGAGTCAGGC-3′, RV 5′-GAACAGGTCGGACATCACCA-3′ [100]), and 18S rRNA (FW 5′-GTAACCCGTTGAACCCCATT-3′, RV 5′-CCATCCAATCGGTAGTAGCG-3′ [101]) was used as a reference gene.

Data were elaborated by the SDS 1.3.1 software (Applied Biosystems, Foster City, CA, USA) and expressed as the threshold cycle (Ct). The fold increase in mRNA expression compared with the control cells not pre-treated and not exposed to H_2_O_2_ and CGA was determined using the 2^−ΔΔCt^ method.

### 4.10. Glucose Uptake Assay

Glucose uptake was determined using the fluorescent D-glucose analog 2-[N-(7-nitrobenz-2-oxa-1,3-diazol-4-yl) amino]-2-deoxy-D-glucose (2-NBDG), followed by the detection of cell-produced fluorescence according to the protocol described by Molonia et al. [102], with slight modifications. The fluorescence intensity was measured using a fluorescence microplate reader (GloMax^®^ Discover System—TM397) (excitation 465 nm, emission 540 nm). The measured signal, proportional to glucose uptake, was normalized to the total protein content and expressed as the relative fluorescence intensity per mg of protein, with values reported in comparison to untreated controls.

### 4.11. Statistical Analysis

Experiments were performed in triplicate and replicated three times. Data are expressed as the mean ± S.D. from three independent experiments. Statistical analysis was carried out by a one-way ANOVA test, followed by Tukey’s HSD, using the software ezANOVA (https://people.cas.sc.edu/rorden/ezanova/index.html accessed on 6 November 2025). Statistical significance was set at *p* < 0.05 and *p* < 0.01. Exact *p*-values are available upon request.

## 5. Conclusions

In conclusion, the results underscore the potential of CGA as an important agent capable of reducing adipocyte senescence, thus contributing to the restoration of adipose tissue homeostasis, and they lay the foundation for future strategies aimed at integrating CGA into nutritional or pharmacological interventions to counteract adipose tissue dysfunction associated with the accumulation of senescent cells. Although our findings provide novel insights into the senotherapeutic potential of CGA, this study was limited to an in vitro model. Further studies, using in vivo models and translational approaches, will be essential to confirm these findings and clarify the therapeutic relevance of CGA in senescence-related metabolic disorders.

## Data Availability

The data that support the findings of this study are available on reasonable request from the corresponding author (Francesco Cimino).

## Supplementary Materials

The following supporting information can be downloaded at: https://www.mdpi.com/article/10.3390/molecules31010167/s1, Figure S1. SA-β-Gal Activity and Lamin B1 Expression. Figure S2. Effect of CGA on Cell Cycle Checkpoint Pathways. Figure S3. Effect of CGA on cell proliferation. Figure S4. Effect of CGA on Oxidative Stress, Inflammatory Signaling and ECM-Remodeling Factors. Figure S5. Effect of CGA on Insulin Signaling Pathway. Figure S6. Effect CGA on Adipogenesis.
