# Supplementary material for: Effects of Chlorogenic Acid on Cellular Senescence in an In Vitro Model of 3T3-L1 Murine Adipocytes
Source: Molecules. 2026 Jan 1;31(1):167. doi: 10.3390/molecules31010167 (PMC12787804; doi:10.3390/molecules31010167)
Supplement: Supplementary file 1 [file molecules-31-00167-s001.zip › molecules-3983350-supplementary.pdf]

Supplementary material

Effect of Chlorogenic Acid on Cellular Senescence in an *In Vitro* Model of 3T3-L1 Murine Adipocytes

Maria Sofia Molonia[a§], Federica Lina Salamone[ab§], Santi Trischitta[a], Antonella Saija[a], Francesco Cimino[a]*†and Antonio Speciale[a]†

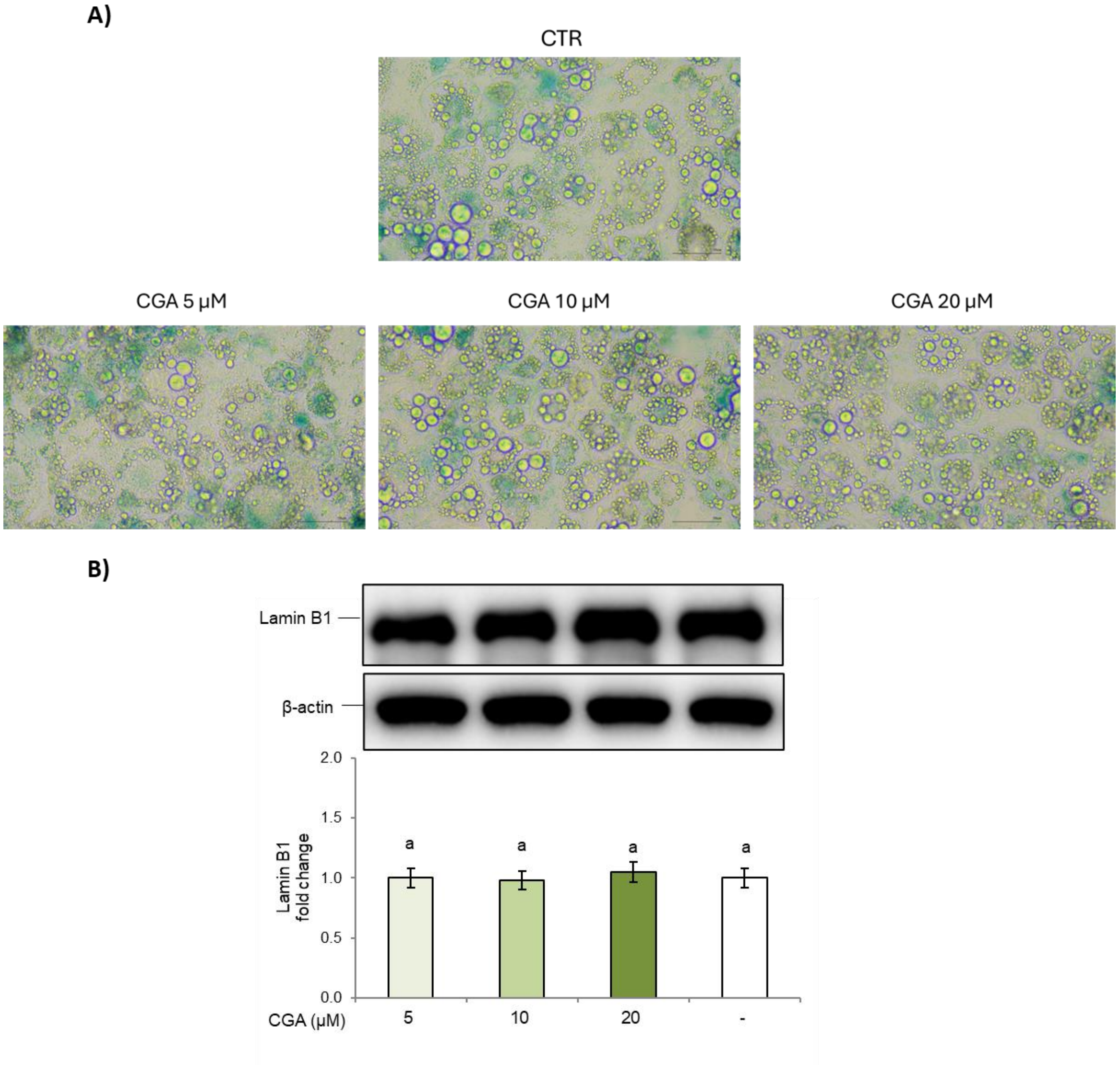

Figure S1. *SA-β-Gal Activity and Lamin B1 Expression.* 3T3-L1 adipocytes were treated during the established senescence induction protocol with CGA (5, 10, or 20 μM) from day 5 to day 10. Cells cultured with the differentiation medium containing the CGA vehicle alone (0.1% v/v DMSO) were used as controls (CTR). (A) Representative images of SA-β-Gal staining (40× magnification- scale bar 50 μm). (B) Lamin B1 protein expression was analyzed by western blot. The densitometry results are reported as fold change compared to control cells. The values were normalized to the

corresponding β-actin value. All results are reported as mean ± S.D. of three independent experiments (n = 3 biological replicates). Means with the same letter are not significantly different from each other (p > 0.05).

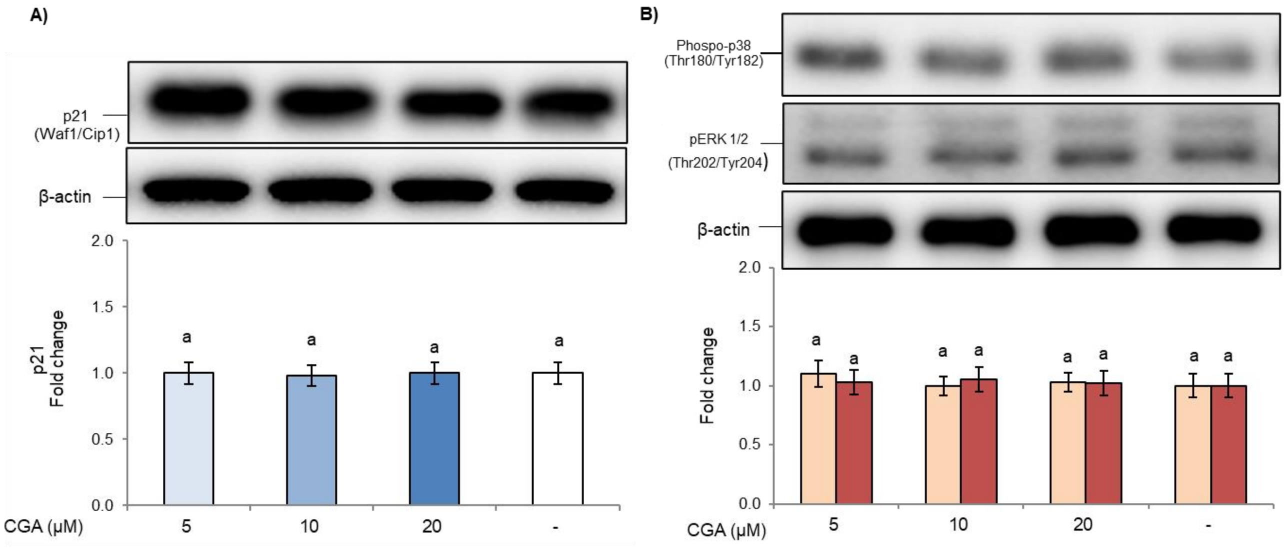

Figure S2. *Effect of Chlorogenic Acid on Cell Cycle Checkpoint Pathways.* 3T3-L1 adipocytes were treated during the established senescence induction protocol with CGA (5, 10, or 20 μM) from day 5 to day 10. Cells cultured with the differentiation medium containing the CGA vehicle alone (0.1% v/v DMSO) were used as controls. (A, B) p21, phosphorylated p38 and pERK 1/2 proteins expression was analyzed by western blot. The densitometry results are reported as fold change compared to control cells. The values were normalized to the corresponding β-actin value. All results are reported as mean ± S.D. of three independent experiments. Means with the same letter are not significantly different from each other (p > 0.05).

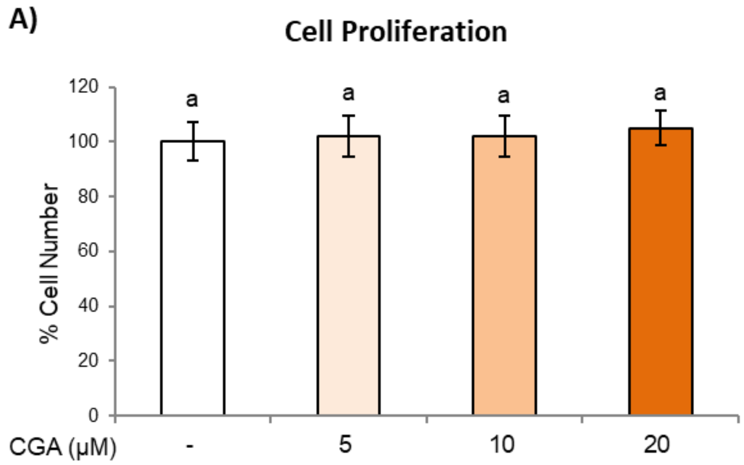

Figure S3. *Effect of Chlorogenic Acid on cell proliferation.* 3T3-L1 adipocytes were treated during the established senescence induction protocol with CGA (5, 10, or 20 μM) from day 5 to day 10. Cells cultured with the differentiation medium containing the CGA vehicle alone (0.1% v/v DMSO) were

used as controls. (A) Cell proliferation was determined by hemocytometer counts and results are expressed as percentage relative to control. All results are reported as mean ± S.D. of three independent experiments (n = 3 biological replicates). Means with the same letter are not significantly different from each other ($p > 0.05$).

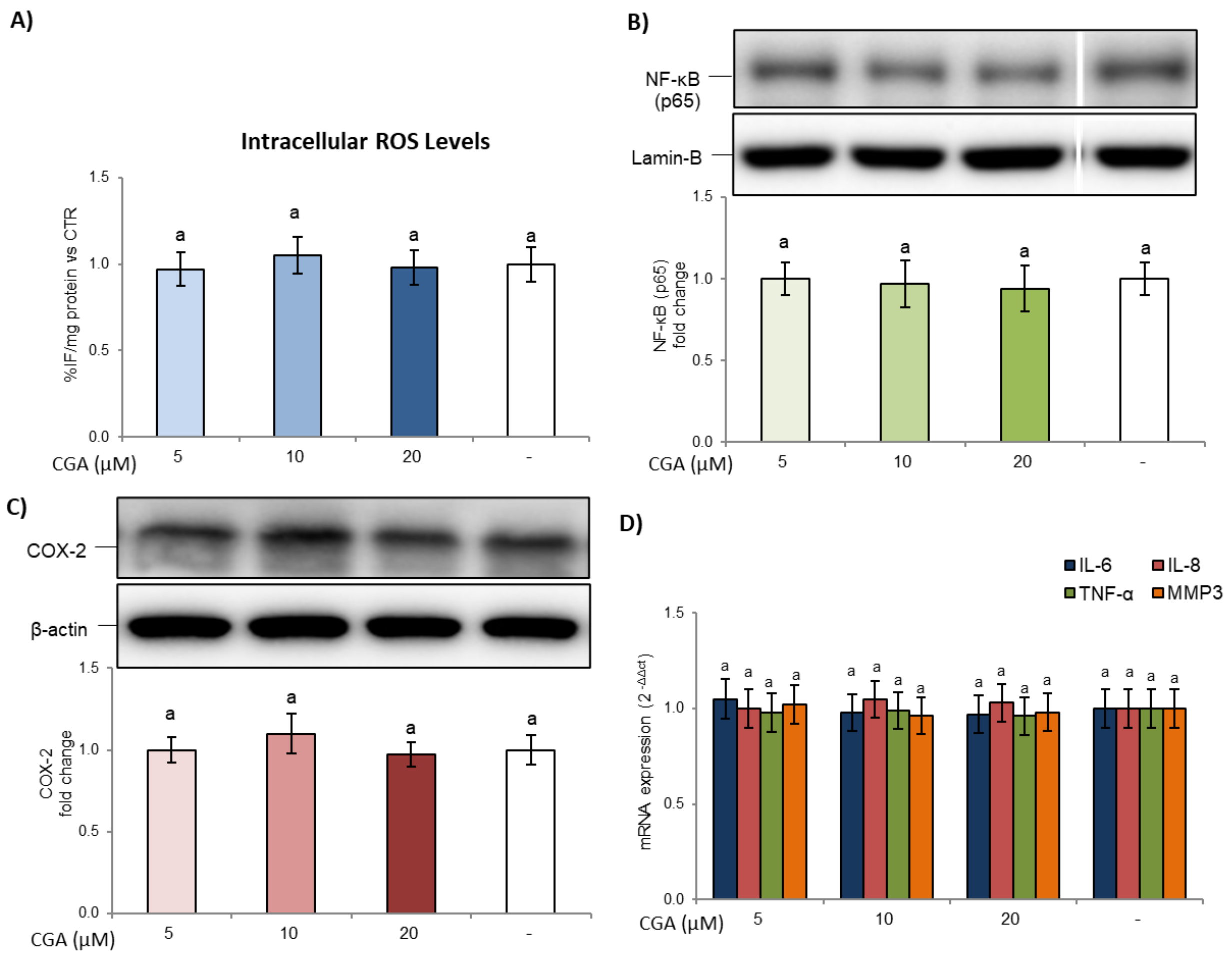

Figure S4. *Effect of Chlorogenic Acid on Oxidative Stress, Inflammatory Signaling and ECM-Remodeling Factors.* 3T3-L1 adipocytes were treated during the established senescence induction protocol with CGA (5, 10, or 20 µM) from day 5 to day 10. Cells cultured with the differentiation medium containing the CGA vehicle alone (0.1% v/v DMSO) were used as controls (CTR). (A) Intracellular ROS levels are reported as % change of fluorescence intensity/mg of proteins against controls. (B, C) Nuclear NF-κB (p65) and COX-2 protein expression were analyzed by western blot. The band of NF-κB (p65) was cropped from original western blot image for illustration purposes; the uncropped images are available in the supplementary material. The densitometry results are reported as fold change compared to control cells; values were normalized to the corresponding Lamin-B and β-actin value. (D) IL-6, IL-8, TNF-α and MMP3 gene expression values are expressed as $2^{-\Delta\Delta Ct}$ and normalized against control cells. 18S rRNA was used as a housekeeping gene. All results are reported as mean ± S.D. of three independent experiments (n = 3 biological replicates). Means with the same letter are not significantly different from each other ($p > 0.05$).

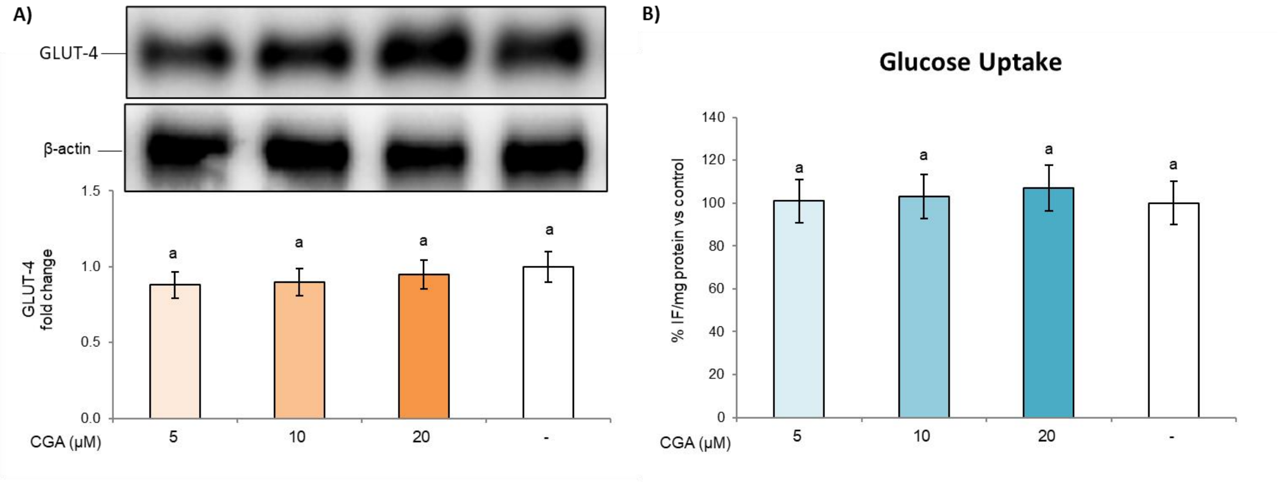

Figure S5. *Effect of Chlorogenic Acid on Insulin Signaling Pathway.* 3T3-L1 adipocytes were treated during the established senescence induction protocol with CGA (5, 10, or 20 μM) from day 5 to day 10. Cells cultured with the differentiation medium containing the CGA vehicle alone (0.1% v/v DMSO) were used as controls. (A) The expression of GLUT-4 protein was analyzed by western blot. The densitometry results are reported as fold change compared to control cells. The values were normalized to the corresponding β-actin value. (B) Glucose uptake results are reported as % change of fluorescence intensity/mg of proteins against control. All results are reported as mean ± S.D. of three independent experiments (n = 3 biological replicates). Means with the same letter are not significantly different from each other ($p > 0.05$).

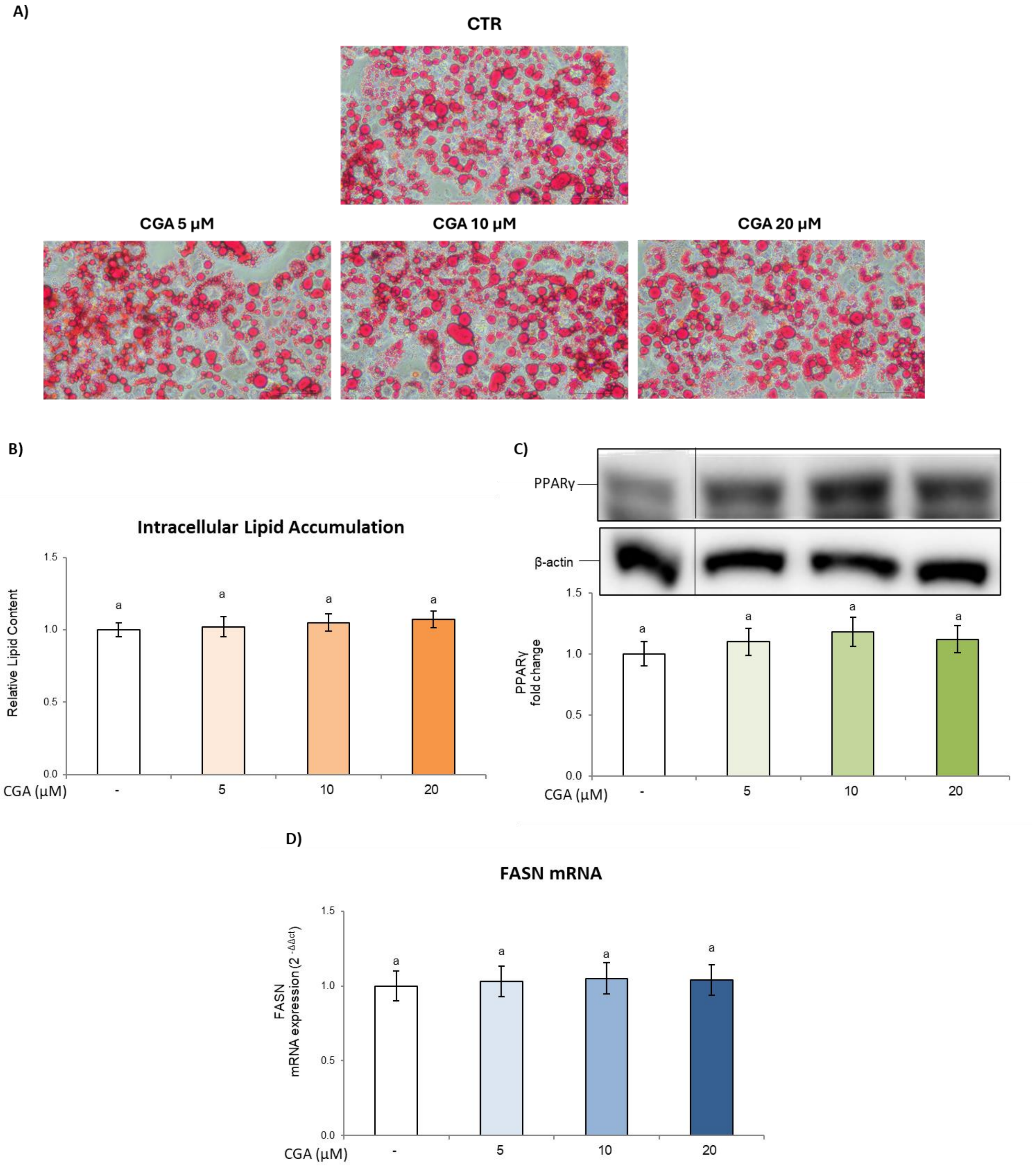

Figure S6. *Effect of Chlorogenic Acid on Adipogenesis.* 3T3-L1 adipocytes were treated during the established senescence induction protocol with CGA (5, 10, or 20 µM) from day 5 to day 10. Cells cultured with the differentiation medium containing the CGA vehicle alone (0.1% v/v DMSO) were used as controls. (A) Representative images of Oil red O staining (original magnification at × 40-scale bar 50 µm) in control cells (CTR) and in cells treated or not with CGA (B) Cell lipid accumulation was expressed as relative content vs control cells. (C) PPARγ protein expression was analyzed by western blot. The densitometry results are reported as fold change compared to control cells. The values were normalized to the corresponding β-actin value. Bands were cropped from

original western blot image for illustration purposes; the uncropped images are available in the supplementary material. (D) FASN gene expression value was expressed as $2^{-\Delta\Delta Ct}$ and normalized against control cells. 18S rRNA was used as a housekeeping gene. All results are reported as mean ± S.D. of three independent experiments (n = 3 biological replicates). Means with the same letter are not significantly different from each other ($p > 0.05$).
